# Structural Basis for the Inhibition of a Phospholipase A_2_-Like Toxin by Caffeic and Aristolochic Acids

**DOI:** 10.1371/journal.pone.0133370

**Published:** 2015-07-20

**Authors:** Carlos A. H. Fernandes, Fábio Florença Cardoso, Walter G. L. Cavalcante, Andreimar M. Soares, Maeli Dal-Pai, Marcia Gallacci, Marcos R. M. Fontes

**Affiliations:** 1 Dep. de Física e Biofísica, Instituto de Biociências, UNESP–Universidade Estadual Paulista, Botucatu, São Paulo, Brazil; 2 Instituto Nacional de Ciência e Tecnologia em Toxinas, CNPq, São Paulo, São Paulo, Brazil; 3 Dep. de Farmacologia, Instituto de Biociências, UNESP–Universidade Estadual Paulista, Botucatu, São Paulo, Brazil; 4 Fundação Oswaldo Cruz (FIOCRUZ), Porto Velho, Rondônia, Brazil; 5 Centro de Estudos de Biomoléculas Aplicadas, Universidade Federal de Rondônia, Porto Velho, Rondônia, Brazil; 6 Dep. de Morfologia, Instituto de Biociências, UNESP–Universidade Estadual Paulista, Botucatu, São Paulo, Brazil; Weizmann Institute of Science, ISRAEL

## Abstract

One of the main challenges in toxicology today is to develop therapeutic alternatives for the treatment of snake venom injuries that are not efficiently neutralized by conventional serum therapy. Venom phospholipases A_2_ (PLA_2_s) and PLA_2_-like proteins play a fundamental role in skeletal muscle necrosis, which can result in permanent sequelae and disability. This leads to economic and social problems, especially in developing countries. In this work, we performed structural and functional studies with Piratoxin-I, a Lys49-PLA_2_ from *Bothropspirajai* venom, complexed with two compounds present in several plants used in folk medicine against snakebites. These ligands partially neutralized the myotoxic activity of PrTX-I towards binding on the two independent sites of interaction between Lys49-PLA_2_ and muscle membrane. Our results corroborate the previously proposed mechanism of action of PLA_2_s-like and provide insights for the design of structure-based inhibitors that could prevent the permanent injuries caused by these proteins in snakebite victims.

## Introduction

In Asia, Africa and Latin America,approximately 98% of the world’s snakebites occur, with 421,000 envenomations and 20,000 deaths by ophidian accidents [1]. However, these numbers may be as high as 1,841,000 envenomations and 94,000 deaths per year, considering the under-reporting that occurs in these regions [1]. The mortality caused by snakebites ishigher thanseveral neglected tropical diseases, including dengue hemorrhagic fever, leishmaniasis, cholera, schistosomiasis and Chagas disease [2]. Consequently, the World Health Organization (WHO) recognizes snakebites as an important neglected tropical disease.

In Latin America, snakes of the *Bothrops*genusare responsible for approximately 80% of all ophidian accidents[3,4]. Envenomation by these snakes is associated with prominent local tissue damage characterized by swelling, blistering, hemorrhaging and necrosis of the skeletal muscle. These effects are not efficiently neutralized by conventional serum therapy and can result in permanent sequelaeand disability[5,6]. The main toxins involved in tissue-damaging activities are the phospholipases A_2_(PLA_2_s)and metalloproteinases, which are the most abundant components of venoms from the *Bothrops*genus[7]. A subgroup of PLA_2_s, known as PLA_2_-like proteins,is catalytically inactive due to lack of Ca^2+^ coordination related to natural mutationsof its primary structure; however, it is still able to induce a drastic local myonecrosis[8–10]. The most studied PLA_2_-like proteins are the Lys49-PLA_2_s, in which the mutations Asp49→Lys and Tyr28→Asn impair the Ca^2+^ coordination[11,12]. Several studies with Lys49-PLA_2_s have shown that segment 115–129 of the C-terminal region, which includes a variable combination of positively charged and hydrophobic residues, is responsible for the myotoxic activity of these proteins[13–16]. Recently, a mechanism of action has been proposed for the Lys49-PLA_2_s and all other PLA_2_-like proteins that includes a quaternary conformational change for the activation of these proteins and the participation of two independent interaction sites with the membrane [17,18].

In folk medicine, especially in developing countries, several vegetal species are employed for the treatment of ophidian envenomations incommunities that lack prompt access to serum therapy [19,20]. In recent years, some studieshave investigated the effects of plants on snakebites, includingthe isolation and characterization of their active constituents and theelucidation of their possible mechanisms of action [19,21–23].

Aristolochicacid (AA) and caffeic acid (CA) are plant compounds with anti-snake venom properties that are used in folk medicine [19–21]. AA (8-methoxy-6-nitrophenanthro (3,4-d-1,3-dioxole-5-carboxylic acid) is an alkaloid found in several *Aristolochia* species. This plant is among the most popular anti-snake venom folk compoundsable to neutralize rattlesnake venomactivity[20]. AA causes a dose-dependent inhibition of *in vitro* phospholipid hydrolysis by human synovial fluid PLA_2_ and snake venomPLA_2_s [24–27]. CA (3-(3,4-dihydroxyphenyl 2-propenoic acid) is a cinnamic acid derivative, abundant in nature and with exceptional biochemical reactivity. It has a large variety of potential pharmacological effects, such as anti-oxidant, anti-cancer and anti-viral activities [28–30]. CA is found in *Vernoniacondensate* leaves, showing antidote activity against *Bothropsjararaca*venom [19,21]. Additionally, crystalline CA derivatives have been demonstrated to be an antidote against snake venom by oral or parenteral administration [31].

In this work, we report functional (neuromuscular-blocking and muscle-damage activities) and crystallographicexperiments aimingto studythe possible inhibitory effects of AA and CA onPrTX-I, a Lys49-PLA_2_ isolated from *Bothropspirajai* snake venom. Our functional studies indicate that these ligands neutralize the myotoxic activity of PrTX-I but do not present effect on the inhibition of neuromuscular blocking activity. The structural studies demonstrated that both ligands interact withPrTX-I in different regions,corroboratingthe previously proposed myotoxic mechanism for PLA_2_-like proteins.

## Material and Methods

### Protein Purification and Inhibitor Source

PrTX-I was isolated from *Bothropspirajai* snake venom by gelfiltration and ion exchange chromatography techniques, as previously described [32]. Aristolochicacid (AA) and caffeicacid (CA) were purchased from Sigma-Aldrich (St Louis, MO, USA).

### Functional Studies

#### Animals

Institutional Animal Care and Use Committee (Institute of Biosciences–Sao Paulo State University–UNESP) approved this study under the number 033/05. Animal procedures were in accordance with the guidelines for animal care prepared by the Committee on Care and Use of Labor. Adult male mice weighing 25–30g were maintainedunder a 12 h light-dark cyclein atemperature-controlled environment (22±2°C) for at least 10 daysprior to the experiments, with food and water *ad libitum*.

#### Neuromuscular-blocking activity

Mice were euthanizedby cervical dislocation followed by exsanguination. The phrenic nerve-diaphragm muscle preparation was removed and mounted vertically in a conventional isolated organ-bath chamber containing 15 mL of Ringer’s physiological solution of the following composition (mM): NaCl, 135; KCl, 5; MgCl_2_, 1; CaCl_2_, 2; NaHCO_3_, 15; Na_2_HPO_4_, 1; glucose, 11. This solution was bubbled with carbogen (95% O_2_ and 5% CO_2_). The preparation was attached to an isometric force transducer (Grass, FT03) to record the twitch tension. The transducer signal output was amplified and recorded on a computer via a transducer signal conditioner (Gould, 13-6615-50) with an AcquireLab Data Acquisition System (Gould). The resting tension was 5 g. Indirect contractions were evoked by supramaximal pulses (0.2 Hz, 0.5 ms) delivered from an electronic stimulator (Grass, S88K) and applied to the phrenic nerve by means of a suction electrode.

The preparation was stabilized for 45 minutes before the addition of a single concentration of toxin. For inhibition experiments, a fixed amount of PrTX-I dissolved in Ringer’s physiological solution was mixed with AA and CAto obtain a 1:1 and 1:5 (w/w) toxin/inhibitor ratio. At molar ratio terms, it means 1:40 and 1:76 for 1:1 (w/w) toxin/inhibitor ratio for AA and CA, respectively, and 1:200 and 1:380 for 1:5 (w/w) ratio. The mixtures were incubated for 30 minutes at 35±2°C. The control experiments were performed in the absence of toxin or in the presence of inhibitors alone. The degree of protection offered by AA and CA after 90 minutes of contact with the preparation was expressed as a percentage of neuromuscular blockade observed in the presence of the mixture of toxin plus inhibitor relative to the blockade observed in the presence of toxin alone.

#### Muscle-damage activity

After the myographicstudy, the diaphragm muscle was removed from the bath and immersed in Bouin’s fixative, and then processed and embedded in Historesin (Kit Historesin Leica). Histological transverse sections (5 mm thick) were cut out in a microtome and stained with hematoxylin and eosin (HE) prior to examination by light microscopy [33]. Morphological damage was quantified in HE-stained preparations using an Imaging Analysis System (Leica, Qwin). The number of fibers with lesions was expressed as a percentage of the total number of cells (muscle damage index) in three non-overlapping non-adjacent areas of each muscle observed at the same magnification. The degree of neutralization offered by AA and CA was expressed as a percentage of the muscle damage index in the presence of the toxin plus inhibitor relative to that index in the presence of the toxin alone.

#### Statistical analysis

The data are expressed as the mean ± S.E.M. The statistical analysis of the data was carried out using ANOVA complemented by the Tukey-Kramer test. Values of P<0.05 were considered significant.

### Structural studies

#### Crystallization of the complexes PrTX-I/AA and PrTX-I/CA

Co-crystallization experiments were performed with PrTX-I at a concentration of 12 mg.mL^-1^. Crystals of the complexes were obtained by the hanging drop method [34]. AA and CA were dissolved in ultrapure water or 50% (w/v) ethanol, respectively, to give an 8:1 molar ratio of inhibitor:protein in the crystallization drops. The drops consisted of 1 μLprotein solution, 0.2 μLinhibitorsolution and 0.8 μL reservoir solution and were equilibrated against 500 μL of the same reservoir solution. The best crystals were obtained after an optimization process for the native protein crystallization conditions [35];the reservoir solution consisted of 26–30% polyethylene glycol 4000 (PEG 4000), 100 mMTris-HCl pH 8.1–8.5 and 200 mM lithium sulfate, as previously described for the PrTX-I/CA complex [36]. Crystals were grown at 291 K for approximately four weeks for both protein complexes.

#### X-ray data collection and processing

The X-ray diffraction data for all crystals were collected at a wavelength of 1.45 Å using a synchrotron-radiation source (MX2 station, Laboratório Nacional de Luz Síncrotron, LNLS, Campinas, Brazil) and a MAR CCD imaging-plate detector (MAR Research). Crystals were mounted in nylon loops and flash-cooled in a stream of nitrogen gas at 100 K using no cryoprotectant. The data were processed using the HKL program package [37].

#### Structure determination and refinement

The crystal structures were determined by molecular replacement techniques implemented in the program MOLREP [38] from the CCP4i program package [39] using the coordinates fromthe crystal structures of thePrTX-Icomplexed with α-tocopherol (PDB ID 3CYL)[35]and bothropstoxin-I (BthTX-I), and a Lys49-PLA_2_ isolated from *Bothropsjararacussu*venom complexed with PEG 4000 (PDB ID 3IQ3) [12]as models forthe PrTX-I/AAandPrTX-I/CA complexes, respectively. The modeling processes were always performed by manual rebuilding with the program Coot[40]usingelectron density maps calculated with the coefficients 2|F_obs_|-|F_calc_|. The models were improved, as judged by the free R-factor [41], through rounds of crystallographic refinement (positional and restrained isotropic individual B-factor refinement, with an overall anisotropic temperature factor and bulk solvent correction) usingPHENIX[42]. In the structure of the PrTX-I/AA complex, due to a lack of electron density, part or full side chains of the following residues were excluded: Leu2 (monomer A), Phe3 (monomers A and B), Lys7(A,B), Lys11(A), Lys15 (A,B), Lys20(A), Val31(A), Leu32(A,B), Lys36(A,B), Arg43(A), Lys53(A,B), Lys57(A,B), Lys69(A,B), Lys70(A,B), Arg72(A,B), Lys78(A,B), Asp79(A), Asn88(A), Glu94(B), Lys115(A), Lys116(A), Lys122(A), Phe125 (A,B), Lys127(A,B), Lys129(B), and Asp130(A). In the structure of the PrTX-I/CA complex, part or full side chains of the following residues were excluded: Phe3(A), Leu32(B), Lys36(A), Lys53(B), Lys57(A,B), Thr59(A), Lys69(A,B), Lys70(A,B), Lys78(A), Lys127(B), and Lys129(A). Thestereochemicalqualities of the models weredetermined with the PHENIX and MolProbity programs[42,43].

## Results

### Neuromuscular blocking activity

**Fig 1:**
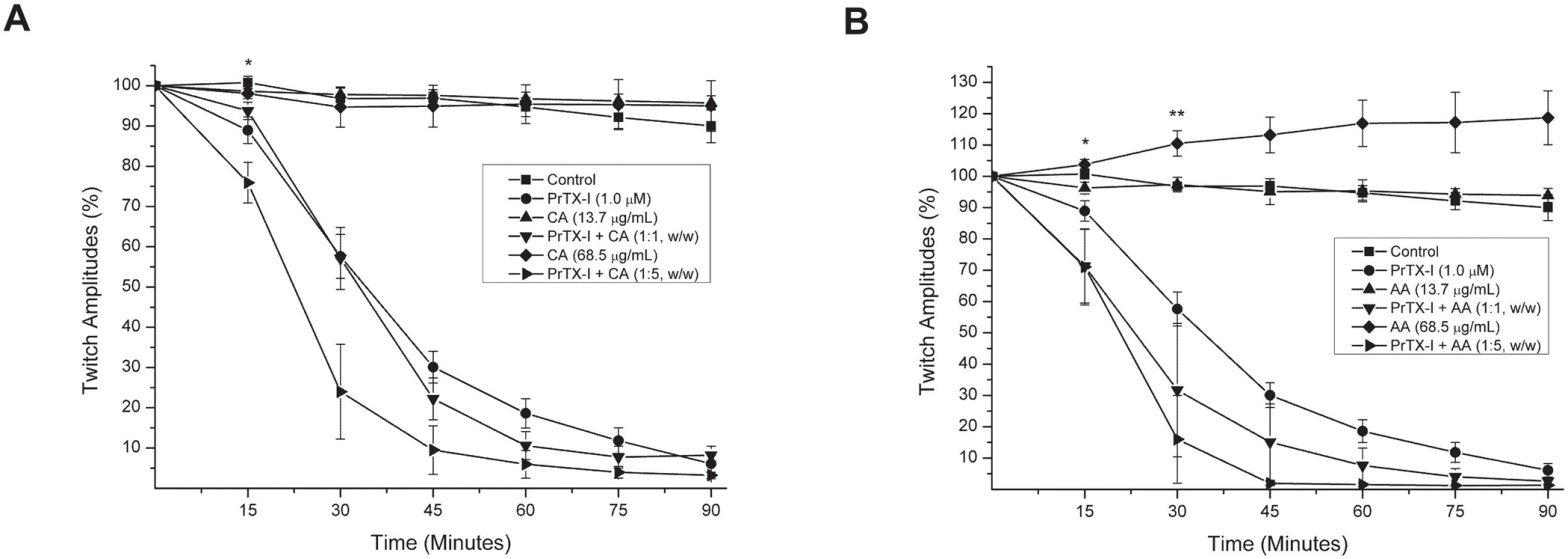
Effects of PrTX-I and PrTX-I pre-incubated with caffeicacid [CA](A) and aristolochicacid[AA] (B) on indirectly evoked twitches in mouse phrenic-diaphragm preparations. The ordinate represents the percentage amplitude of twitches relative to the initial amplitude. The abscissa indicates the time from the beginning of each treatment in the organ bath. The points are the mean ± S.E. * indicates the point at which differences between PrTX-I treatments (alone and pre-incubated with CA and AA) and the control become significant. ** indicates the point at which differences between AA (68.5 μg/mL) and the control become significant.

PrTX-I (1.0 μM) promoted a time-dependent blockade of indirectly evoked twitches in mice phrenic-diaphragm preparations. After 90 minutes, the twitch amplitudes were reduced to 89.4% (Fig 1). The paralyzing effect of PrTX-I could not be reversed by washing the preparation for at least 30 minutes with toxin-free physiological solution (data not shown). The mean time required to reduce the twitch amplitudes by 50% (T_½_) was 34.0 ± 2.4 minutes. Pre-incubation with CA (1:1 and 1:5 w/w) or AA (1:1 and 1:5 w/w) did not prevent the neuromuscular blockade induced by PrTX-I (Fig 1A and 1B). Alone, CA did not affect the twitch amplitude(Fig 1A),whileAA(68.5 μg/mL) promoted a facilitator effect (Fig 1B).

### Muscle-damaging activity

**Fig 2:**
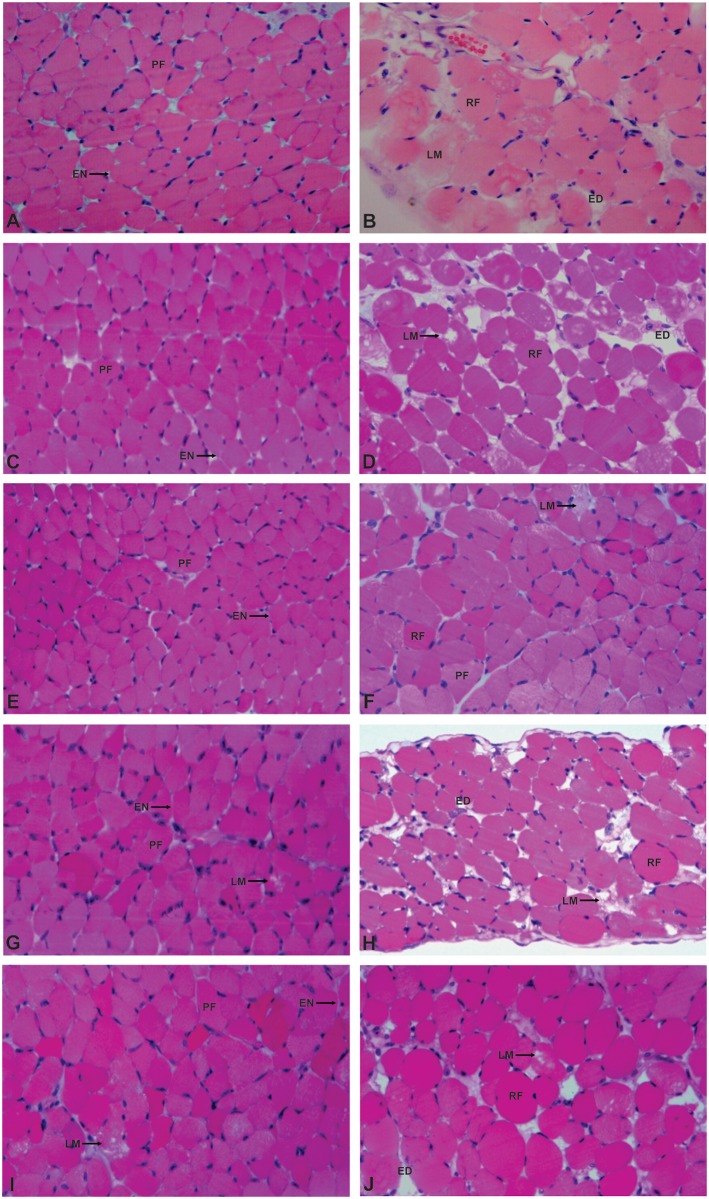
Light micrographs of mouse diaphragm muscles submitted to hematoxylin and eosin staining. Control muscle (A) and muscle exposed to caffeicacid (CA) and aristolochic acid (AA) (C, E, G and I) show fibers with normal appearance as evidenced by the polygonal aspect of fibers (PF) and endomysium (EN). A few fibers present loss of myofibrils in the muscle exposed to AA (G and I). (B) Muscle exposed to PrTX-I: edema (ED), round fibers (RF), some of which present loss of myofibrils (LM). (D, F, H and J) Muscle exposed to PrTX-I pre-incubated with CA and AA: The fibers have characteristics observed less frequently in the fibers treated with the PrTX-I alone, except in J, which occurred at similar frequencies.

**Fig 3:**
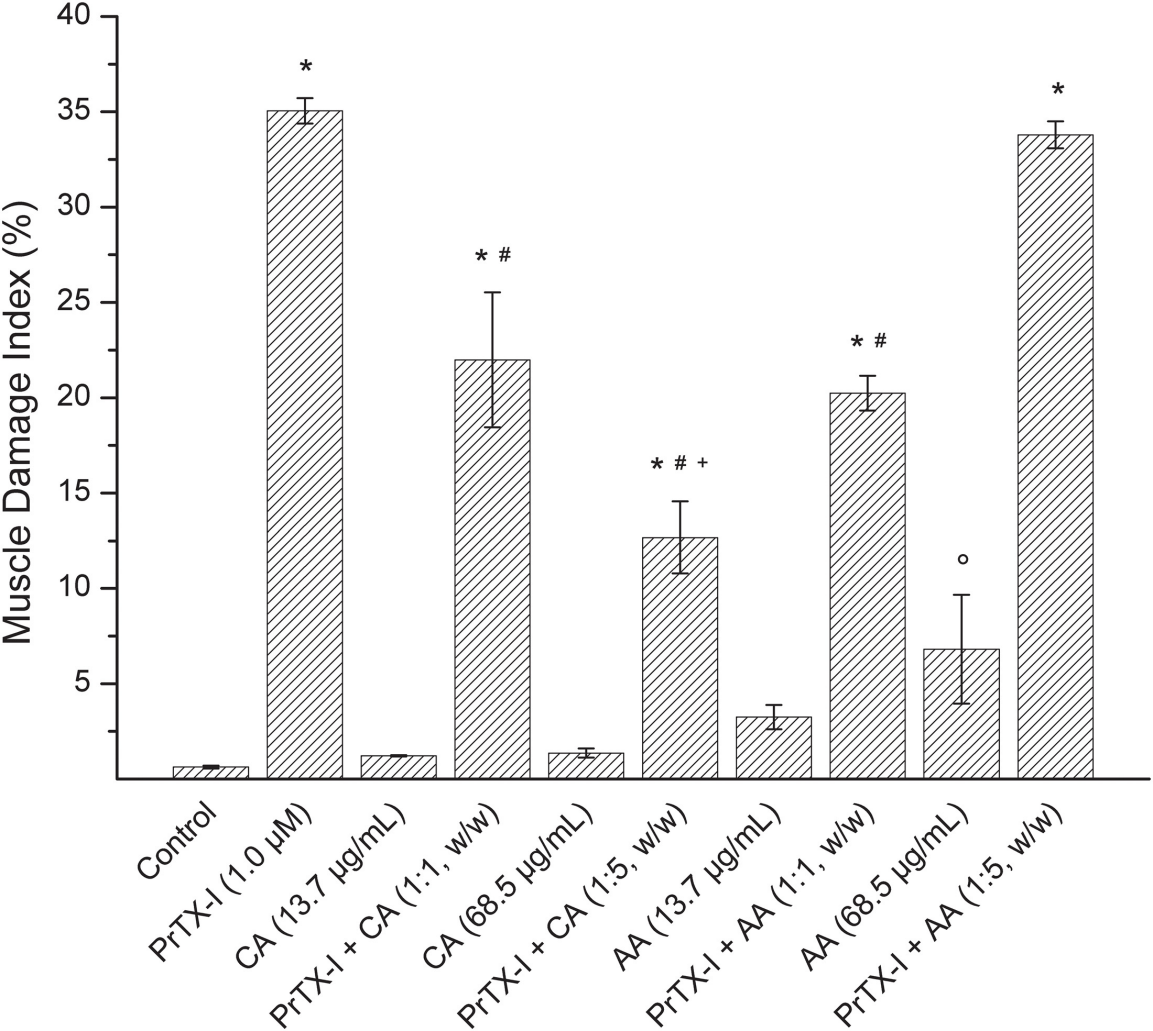
Effect of CA and AA upon the muscle damage index induced by PrTX-I in mouse diaphragm preparations. The ordinate represents the percentage of damaged fibers relative to normal fibers and the abscissa indicates the experimental groups. The bars are expressed as the mean ± S.E. * indicates when differences between PrTX-I treatments (alone and pre-incubated with CA or AA) and their respective controls was significant. # indicates when there were significant differences between PrTX-I pre-incubated with inhibitors and PrTX-I alone treatments. ^+^ indicates significant differences between PrTX-I treatments pre-incubated with CA. ° indicates significant differences between the AA (68.5 μg/mL) treatment and the Control group.

Light microscopy showed that control and CA or AA-treated muscles were of normal appearance. Fibers were delimited by a thin layer of connective tissue (endomysium) and presented a polygonal shape, with an acidophilic sarcoplasmand peripheral nuclei(Fig 2A, 2C, 2E, 2G and 2I). A few fibers from muscles exposed to AA (13.7 μg/mL and 68.5 μg/mL) revealed a loss of myofibrils (Fig 2G and 2I). However, as shown in Fig 3, only muscles exposed to AA (68.5 μg/mL)had a significantmuscle damage index compared to the control (6.8 ± 2.9%, n = 3*vs*.1.2 ± 0.1%, n = 4). After 90 minutes of contact with PrTX-I, the diaphragm muscle showed several changes, such as round fibers and edema. Many fibers presented cytoplasm areas devoid of myofibrils, some with a central nucleus(Fig 2B). The muscle damage index for the PrTX-I group was significantly higher than the control (35.1 ± 0.7%, n = 4*vs*1.2 ± 0.1%, n = 4)(Fig 3). In contrast, pre-incubation with CA (13.7 μg/mL and 68.5 μg/mL) or AA (13.7μg/mL) reduced the myotoxic effects of PrTX-I. In fact, diaphragm muscles exposed to the pre-incubation product presented a more conserved aspect (Fig 2D, 2F and 2H). The muscle damage indices of these preparations were 22.0 ± 3.5% and 12.7 ± 1.9% (PrTX-I/CA at ratios of 1:1 and 1:5, respectively) and 20.2 ± 0.9% (PrTX-I/AA at 1:1) (Fig 3). On the other hand, the pre-incubation of AA with PrTX-I at the ratio 1:5 (w/w) did not reduce the muscle damage index when compared to PrTX-I alone (33.8 ± 0.7%, n = 3*vs*. 35.1 ± 0.7, n = 4) (Fig 2J).

### Overall crystallographic structures

**Fig 4:**
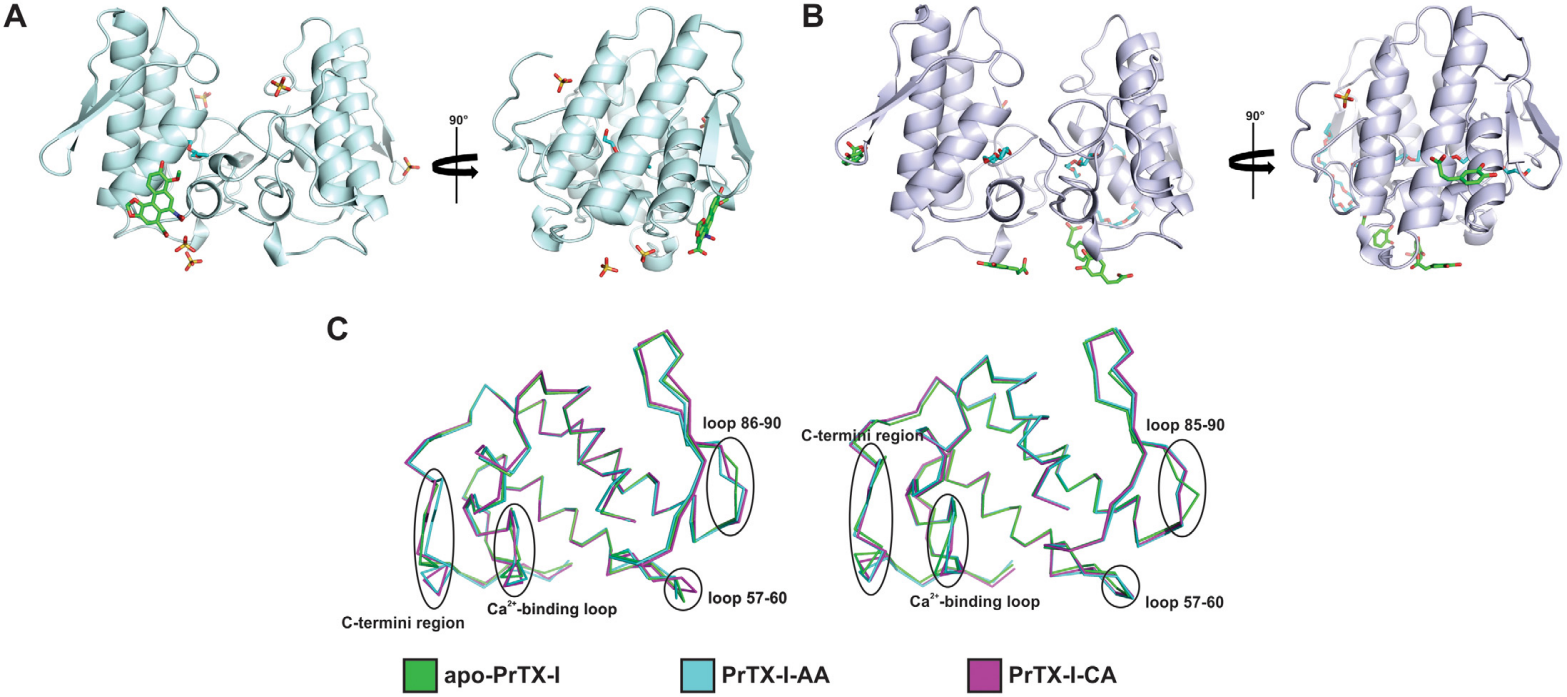
Dimeric structures of (A) PrTX-I complexed to aristolochic acid (PrTX-I/AA) and (B) PrTX-I complexed to caffeic acid (PrTX-I/CA) shown as a cartoon representation. PEG molecules, sulfate ions,AA and CAare indicatedby sticks (in cyan, yellow, blue and green, respectively). In yellow sticks are also highlighted the aminoacids that compose MDiS (Leu121)andMDoS (Lys20, Lys155, Arg118) regions, which interact with AA and CA, respectively. (C) C_α_ superposition of apo-PrTX-I, PrTX-I/AA, PrTX-I/CA and PrTX-I complexed to rosmarinic acid (PrTX-I/RA) (monomers A and B, respectively) highlighting the most important structural deviations between them.

Crystals of both complexesdiffracted at high resolution (Table 1) and classified as belonging to the P2_1_2_1_2or P2_1_ space groups for PrTX-I/AA and PrTX-I/CA, respectively. The refinements converged to final R values of 17.3% (R_free_ = 23.5%) and 18.3% (R_free_ = 22.9%), respectively, for PrTX-I/AA and PrTX-I/CA. The final models (Fig 4) are of a stereochemical quality expected for structures with the same resolution, as indicated by r.m.s.d bonds, r.m.s.d. angles and Ramachandran plot analyses (Table 1). Both structures have seven disulfide bridges in each monomer with the following structural features: (i) an N-terminal α-helix; (ii) a “short” helix; (iii) a Ca^2+^ binding loop; (iv) two anti-parallel α-helices (2 and 3); (v) two short strands of an anti-parallel β-sheet (β-wing); and (vi) a C-terminal loop (Fig 4), similar to all other class II PLA_2_s [44,45].

**Table 1:** X-ray data collection and refinement statistics.

	PrTX-I-Aristolochic Acid	PrTX-I-Caffeic Acid
Unit Cell (Å)	*a* = 68.3; *b* = 70.9; *c* = 44.0	*a* = 39.2; *b* = 72.8; *c* = 44.6;β = 102.1°
Space Group	P2_1_2_1_2	P2_1_
Resolution (Å)	25.61–1.96 (2.03–1.96)^a^	37.34–1.65 (1.70–1.65)^a^
Unique reflections	15848 (1541)^a^	27814 (2724)^a^
Completeness (%)	99.22 (98.59)^a^	94.47 (92.59)^a^
R_merge_ ^b^	6.3 (49.0)^a^	6.5 (39.5)^a^
Mean I/σ (I)	14.33 (2.02)^a^	27.4(2.34)^a^
R_cryst_ ^c^(%)	17.30	18.23
R_free_ ^d^(%)	23.52	22.87
Number of non-hydrogen atoms^e^		
Protein	1749	1849
Ligands	60	108
Waters	174	289
RMS (bonds)^e^	0.007	0.008
RMS (angles)^e^	1.14	1.18
Average B-factor (Å^2^)^e^		
Protein	29.60	32.10
Ligands	54.40	56.40
Solvent	37.10	40.60
Ramachandran favored (%)^e^	98	95
Ramachandran outliers (%)^e^	0	0
Clashscore^f^	4.77	11.37
MolProbity Overall Score^f^	1.54	1.78

^a^ Numbers in parenthesis are for the highest resolution shell.

^b^ R_merge_ = ∑_hkl_(∑i(|I_hkl,i_-<I_hkl_>I))/∑_hkl,i_<I_hkl_>, where I_hkl,i_ is the intensity of an individual measurement of thereflection with Miller indices h, k and l, and <Ihkl> is the mean intensity of that reflection. Calculated for I>-3σ (I).

^c^ R_cryst_ = ∑_hkl_(||Fobs_hkl_|-|Fcalc_hkl_||)/|Fobs_hkl_|, where |Fobs_hkl_| and |Fcalc_hkl_| are the observed and calculated structure factor amplitudes, respectively.

^d^ R_free_is equivalent to R_cryst_ but calculated with reflections (5%) omitted from the refinement.

^e^ Calculated with Phenix [42].

^f^ Calculated with MolProbity[43].

**Fig 5:**
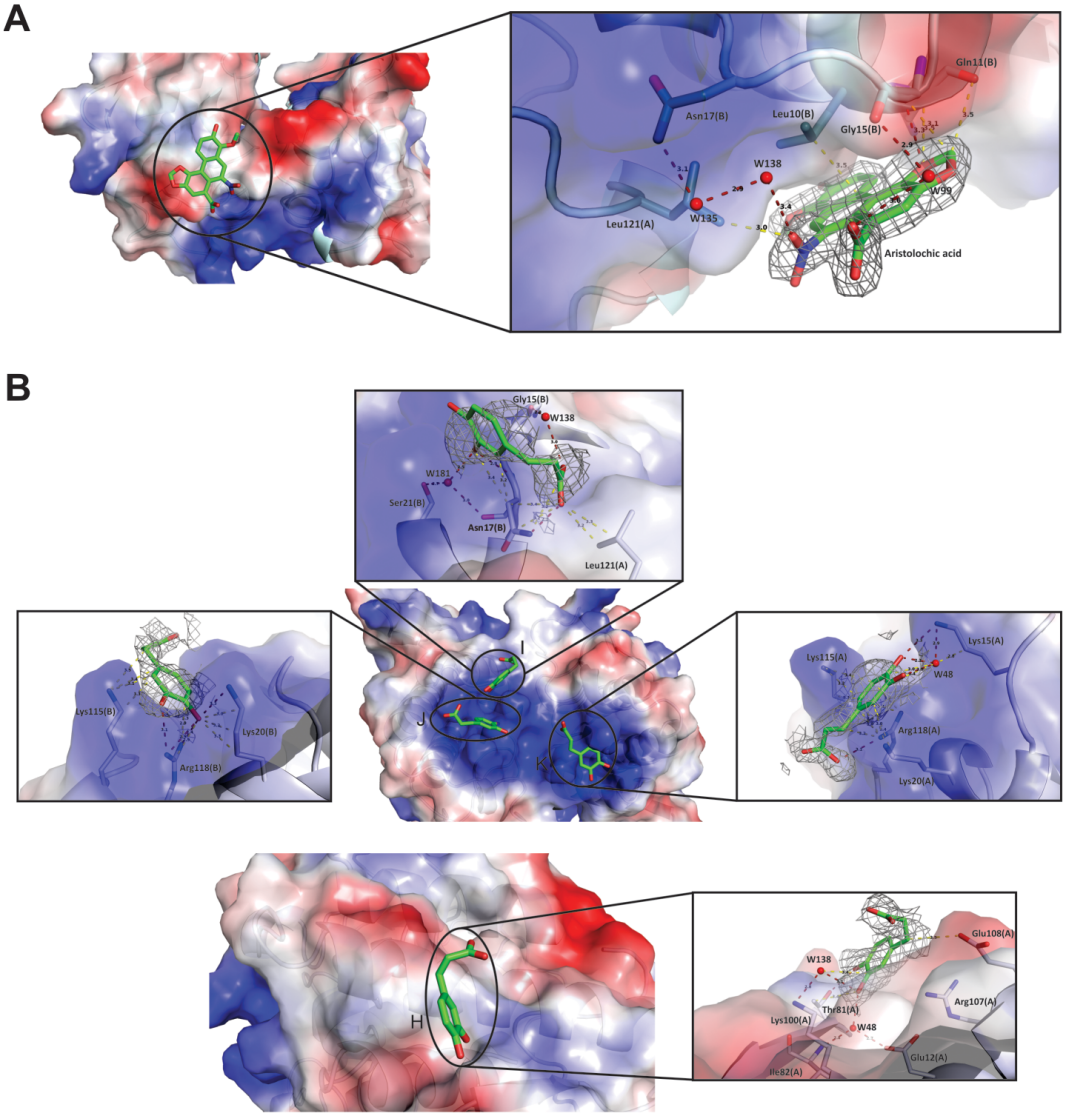
Electrostatic potential surface of(A) PrTX-I complexed to aristolochic acid (PrTX-I/AA) and (B) PrTX-I complexed to caffeic acid (PrTX-I/CA) crystal structures. The distances (Å) between protein inhibitorsare shownas yellow dashes for bump distances and red dashes for hydrogen bond distances. Residues of monomers A and B in contact with AAand CAare represented by sticks. Omit electron density maps for AA and CA molecules (gray meshes) were calculated with the coefficients 2|F_obs_|-|F_calc_| contoured at 1.0 standard deviation.

**Fig 6:**
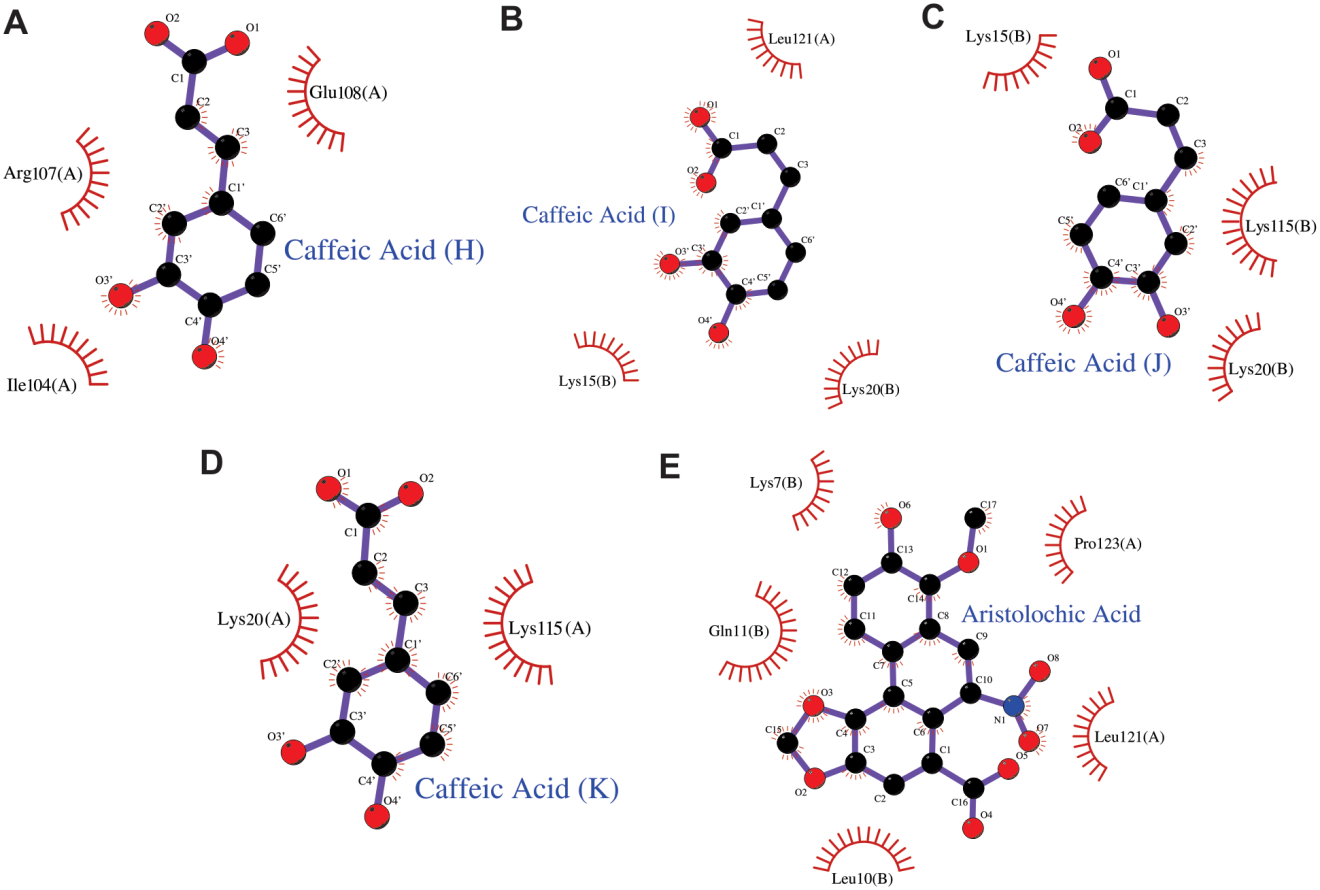
Hydrophobic contacts between caffeic acid (A, B, C and D) and aristolochic acid (E) molecules with PrTX-I observed in crystal structures. Drawn using Ligplot[60].

The inspection of the 2ǀF_obs_ǀ-ǀF_calc_ǀ electronic density mapof the PrTX-I/CA structure revealed the presence of four CA molecules establishing hydrogen bonds in monomer A with Lys15 and Arg118 and by water molecules with Ile82 and Lys100 (Fig 5). In monomer B, CA molecules establish hydrogen bonds with Lys20 and Arg118 by water molecules with Gly15, Asn17 and Ser21 (Fig 5). Moreover, CA molecules establish hydrophobic contacts with Lys20, Lys115, Ile104, Arg107, Glu108 and Leu121 in monomer A and with Lys15, Lys20 and Lys115 in monomer B (Fig 6). In other Lys49-PLA_2_s crystal structures, sulfate ions were found interacting with most of the residues that establish contacts with CA on the PrTX-I/CA structure [18,35]. However, it is possible to determine, by checking electron density omit-maps, an unambiguous interpretation of CA for these maps (Fig 5). Moreover,CA molecules modeled on these maps presented lower mean B-factor values (55.7 Å^2^) when compared with sulfate ions modeled on the same region (88.9 Å^2^). For comparison reason, sulfate ions modeled in the same regionfor other Lys49-PLA_2_s structures presented following B-factor values: 44.2 Å^2^inPrTX-I/αT (PDB ID 3CYL); 68.8 Å^2^ in BthTX-I/αT (PDB ID 3CXI); and 52.9 Å^2^ in MTX-II/PEG4K (PDB ID 4K06).

In addition,three PEG 4000 moleculeswere noted in the PrTX-I/CA structure,with two PEGs inside the hydrophobic channels from both monomersand the third PEG moleculeinteracting with Lys7 on monomer B. The PEG molecules found in these sites are found in other Lys49-PLA_2_s structures [12,18,46]. Finally, in the PrTX-I/CA structure, a sulfate molecule establishedhydrogen bonds with Arg33 in monomer A, similarly to sulfate ions found in other Lys49-PLA_2_s structures [18,35].

On the other hand, in the PrTX-I/AA structure just one AA molecule was observedwhich established hydrogen bonds with N-terminalresidues from monomer B (Gly15 and Asn17 by two water molecules)in close proximity to the C-terminal region from monomer A, especially Leu121 (Fig 5). Moreover, AA established hydrophobic contacts withLeu121 and Pro123 from monomer A and with Lys7, Gln11 and Leu10 from monomer B (Fig 6). Furthermore, fivesulfate ionsinteracted with several basic residues ofthe PrTX-I/AA structure (Lys20, Arg34, Lys115 and Arg118), similarly to that observed in other Lys49-PLA_2_s structures. Finally, a part of a PEG 4000 molecule (nine atoms in the electron density map) inside the monomer B hydrophobic channelwas noted.

### Structural comparison between apo-PrTX-I and complexes with PrTX-I crystal structures

Superposition among the complexed structuresPrTX-I/AA,PrTX-I/CA and PrTX-I rosmarinic acid (PrTX-I/RA) [47]resulted in a Cα atom r.m.s. deviation in the range of 0.3–0.4 Å. In contrast, a similar superposition between thecomplexed structures and the apo structure had anr.m.s.d. close to 1.0 Å (Table 2). These values are comparable to other Lys49-PLA_2_s [35,48], where a pattern was observed among apo forms with an r.m.s.d. of approximately 1.0 Å, while for structures that present with molecules in the hydrophobic channel (e.g., PEG and α-tocopherol)these values are significantly lower. These higher values for apo forms occur mainly due to differences in their C-termini, demonstrating the higher conformational flexibility of this region. The main differences occur in three regions of each monomer: Ca^2+^ binding loop (residues 29–34), the loop after the β-wing (residues 85–90) and in the C-termini (117–130)(Fig 4). These differences are observed on several Lys49-PLA_2_s superpositions[12,18]and thus, the ligands do not cause relevant changes in the tertiary structure conformation of the protein.

**Table 2:** Superposition between protomers of apo-PrTX-I, PrtTX-I complexed to Aristolochic Acid (PrTX-I/AA), PrTX-I complexed to Caffeic Acid (PrTX-I/AA) and PrTX-I complexed to Rosmarinic Acid (PrTX-I/RA) crystallographic structures (r.m.s. deviations (Å) of C_α_ atoms).

		apo-PrTX-I	
		Monomer A	Monomer B
PrTX-I/AA	Monomer A	0.82	0.41
	Monomer B	0.83	0.49
PrTX-I/CA	Monomer A	0.79	0.56
	Monomer B	0.83	0.58
PrTX-I/RA	Monomer A	1.04	0.55
	Monomer B	0.98	0.59

Regarding the quaternary structure, PrTX-Istructures complexed to AA CA or RA have two protomers in the asymmetric unit (Fig 4) and present similar oligomeric structures. As observed for other bothropic Lys49-PLA_2_s [35], there are two choices of dimeric structure in their unit cells. However, there are several examples of experimental and functional data for this class of proteins showing that the oligomeric conformation known as the “alternative dimer” is the most likely to occur in solution(for a review refer to[17]). Then, both PrTX-I structures were refined on this oligomeric conformation. In structures that present molecules in a hydrophobic channel solved in an alternative dimer conformation, it was observed that a Tyr119:Tyr119 interchain hydrogen bond is formed between the monomers[35]. This interactionwas also observed for the complexedPrTX-I structures (AA, CA or RA) because these structures present PEG molecules in their hydrophobic channels. According to Fernandesand colleagues [18], PLA_2_-like structures with molecules at the hydrophobic channel are in their active state because the entrance of a molecule in this channel leads to a quaternary conformational change. This conformational change constitutes the first step of mechanism of action of these proteins[18]. In addition, this phenomenon can be measured by the “two-angle model” previously proposed, which reflects the aperture and torsion of the dimer after molecular binding on hydrophobic channel [35]. PrTX-I/AA,PrTX-I/CA and PrTX-I/RA have 40°,39° and 43° torsion angles and 27°, 28° and 23°aperture angles, respectively. These values are in agreement with structures in the active state [35,48]. In contrast, inactive (or apo) structures have higher values of torsion angles (60°-61°) and smaller values of aperture angles (6°-7°) [35,48].

## Discussion

### The plant-derived molecules caffeic and aristolochic acids inhibit PrTX-I myotoxic activity

Several groups of indigenous people use specific plant extracts against snakebites, and the identification of their active compounds is an active field of study. Recently, some studies showed that several constituents of these plant extracts containanti-snake venom properties [19,21–23]. Recently, structural and functional studies demonstrated that rosmarinic acid (RA) was able to inhibit the *invitro* paralyzing activity caused by PrTX-I in mice neuromuscular preparationsby 80% [47]. In addition, light and electron micrographs of mouse diaphragm muscle also demonstrated that PrTX-I may severely damage muscle fibers [47,49]. On the other hand, preparations exposed to PrTX-I that were pre-incubated with RA presented fibers with normal aspects [47].

In this study, both caffeic (CA) and aristolochic acids (AA) partially neutralized the muscle damage promoted by PrTX-I. As CA is a RA precursor, it was expected that its inhibitory activities against PrTX-I effects would be similar in the phrenic-diaphragm preparation. However, CA was ineffective at inhibiting the *invitro* paralyzing activity of this toxin. On the other hand, light microscopy analysis of muscle preparations submitted to myographic experiments showed that CA protected against the myotoxic effect induced by PrTX-I by 40% and 65% (PrTX-I/CA at ratios of 1:1 and 1:5, respectively). The different results between the CA and RA treatments probably was due to distinct interaction sites of these ligands with PrTX-I (see next two sectionsfor more detailed discussion about ligands binding). Similarly to CA, AA did not inhibit the neuromuscular blockade induced by PrTX-I. At a higher concentration (68.5 μg/mL), AA alone promoted negative effects on the phrenic-diaphragm preparation, where it caused twitches facilitated by an unknown mechanism. In addition, there were injured fibers in these preparations, which may explain the high muscle damage index when the PrTX-I/AA ratio was 1:5. Interestingly, at low concentrations (13.7 μg/mL), there was no such damage and AA promoted a partial protection (43%) to myotoxicity induced by the toxin (morphological analysis). Except for the intrinsic toxic effect of AA on the neuromuscular preparation, the toxin neutralization by AA and CA was similar, even though they interact in different regions of PrTX-I.

The paralyzing and muscle damage activities promoted by myotoxicLys49-PLA_2_s, represented here by PrTX-I, were due to their ability to alter the integrity of muscle cell membranes (for a review see [50]). After the initial binding, these myotoxins interact with sarcolemma and promote its destabilization, altering the permeability to ions (Na^+^, K^+^, Cl^-^ and Ca^2+^) and macromolecules [8,50–53]. The first consequence of the ionic collapse is the cell depolarization and the lack of excitability due to the inactivation of voltage-dependent Na^+^-channels, thus impairing the generation of the action potential along the muscle fibers and determining the muscle paralysis. The continuous depolarization surrounding the endplate also reaches the motor nerve terminal, reducing the magnitude of the nerve action potential and consequently the acetylcholine (ACh) release, contributing to muscle paralysis [50, 54, 55]. Another consequence of the cell depolarization is the release of Ca^2+^ from intracellular pools [56], starting several deleterious effects, such as myofilament hypercontraction, mitochondrial damage and activation of Ca^2+^-dependent proteases and phospholipases [8,51,52], which culminates in the muscle injury [53,57].

Although the paralysis and the muscle fiber damage induced by Lys49-PLA_2_s are triggered by alterations in membrane permeability, the contractile process is more sensitive to such action because of its dependence on the cell excitability. Thus, ligands that partially inhibit the Lys49-PLA_2_ actions on cell membrane, such as CA and AA, promotes a partial protection of myotoxicity (up to 65% and 43%, respectively) with no positive interference on muscle paralysis. Corroborating this hypothesis, we have previously demonstrated that ligands able to promote a more effective binding to Lys49-PLA_2_, as rosmarinic acid, efficiently prevent both effects[47].

### Aristolochic and caffeic acids bind to different regions of PrTX-I and cause myotoxic inhibition by two different mechanisms

Recently, Fernandesand coworkers[18] proposed anactionmechanism for Lys49-PLA_2_s composed of five steps, which includes an allosteric transition and two independent sites of interaction with the target membrane: i)a cationic membrane docking site (MDoS), composed of the basic residues (usually Lys20, Lys115 and Arg118)responsible for the anchorage of the protein on membraneandii)a hydrophobic membrane disruption site (MDiS) composed of hydrophobic residues exposed to solvent (Leu121 and Phe125)responsible for the disruption of the membrane.

Interestingly, inthis study, we demonstrated that both ligands (AA and CA) are boundto residues related to MDoS and MDiS regions of the toxin and this fact may explain theirinhibitory characteristics. In the PrTX-I/CA crystal structure, four CA molecules interact with the protein. Two of them interact with Lys20, Lys115 and Arg118 residues for both monomers by hydrogen bonds and hydrophobic interactions (Figs 5 and 6). In other Lys49-PLA_2_ crystal structures, it is common to observe sulfate ions, whicharise from the crystallization conditions, establishing similar contacts with these residues[18,35]. Based on analysis of sulfate positions in PLA_2_ crystallographic structures,Bahnson*et al*[58]hypothesized that sulfate ions may simulatethe anion head of the phospholipid, revealing putative regions of the protein that could interact with the target membrane. This observation leddos Santos and colleagues[35] to propose a similar hypothesis for Lys49-PLA_2_s, which was corroboratedbyFernandesand colleagues[18], who calledthis region the MDoS. Therefore, these data indicate that CA molecules inhibit PrTX-I myotoxic activity due totheir interaction withthe MDoS region and consequentlyavoid the docking of the toxinwith the membrane. Regarding the other CA molecules, an interaction with Gly15, Asn17, Ser21 and Leu121 residues close to the MDiS region was observed, suggesting that this CA molecule may also bind to the membrane disruption site of PrTX-I. The fourth CA molecule interacts with Lys100 and Glu108 residues butno function has beenassignedto these residues; thus,the binding of this molecule can be attributed to a non-specific interaction.

The crystal structure of PrTX-I/AAreveals an aristolochic acid molecule interacting by hydrogen bonds with Gly15(B) and via water molecules with Asn17(B) and Gln11(B) (Fig 5). This ligandalso interacts with the N-terminal portion of monomer B (Lys7, Leu10 and Gln11)andthe C-terminusof monomer A (Pro123),which isnearthe MDiS region (particularly Leu121). Therefore, based on this structural information, wepropose that the inhibitory feature observed for this ligand is due to the physical obstruction of the MDiSregion that impairs the disruption of the target membrane by the toxin.

The crystal structure of a catalytic PLA_2_(an Asp49-PLA_2_)from *Viperarusselli*venom complexed to aristolochic acid was also solved[27]. Interestingly, this ligand binds in a different region compared to PrTX-I and the catalytic inhibition observed for this protein has adifferent structural basis. In the case of the structure from*V*.*russelli*, the ligand is located at thehydrophobic channel of the protein, establishing hydrogen bonds with His48 and Asp49 and closing the entrance of its hydrophobic channel [27]. The explanation for these binding differences between catalytic and non-catalytic PLA_2_s is probably thespecific amino acid differences between them because these proteins present a high level of secondary and tertiary structural conservation.

In conclusion, the data presented here demonstrate for the first timethe occurrence of two independent sites of interaction between a protein and a membrane target, whichcontributes to the validation of the proposed myotoxic mechanism [17,18].

### How can we effectively inhibit bothropic Lys49-PLA2s?

One of the main challenges intoxicologytodayis to develop therapeutic alternatives to the treatment of snake venom injuries that are not efficiently neutralized by conventional serum therapy. In the case of Latin American snakes, the local myonecrosis caused by PLA_2_ and PLA_2_-like proteins is the main consequence of their envenomation[55]. This effect is poorly neutralized by anti-venom administrationand, in many cases, may lead to injury, including amputation and permanent disability [5,6]. Therefore, it is important to understand the structural basis of local myonecrosis and to create molecular models than can guide the design of efficient inhibitors that could be used to complement conventional serum therapy. Furthermore, these models also could be used to develop new inhibitors for human PLA_2_s, which are involved in several inflammatory processes and present a very conserved tertiary structure in comparison to snake venom PLA_2_s[44].

In this work, we performed structural and functional approaches to provide substantial information about the inhibition of myotoxic Lys49-PLA_2_s using aristolochic and caffeic acids as molecular models. Taking into account the data presented here and previous studies reported in the literature with other ligands, we propose three different means to inhibit the myotoxicity caused by these proteins:

1. physical blocking forphospholipid binding at the hydrophobic channel of the toxin. There are two different ways to achieve this blocking: a)by binding to the putative "active site" region (His48 residue) (e.g., *p*BPB) [59],b) by preventing its occupation (e.g., rosmarinic acid) [47];
2. obstruction of the protein-membrane docking region (MDoS) (e.g., caffeic acid);
3. obstruction of the protein region related to membrane destabilization (MDiS) (e.g., aristolochic acid).

**Fig 7:**
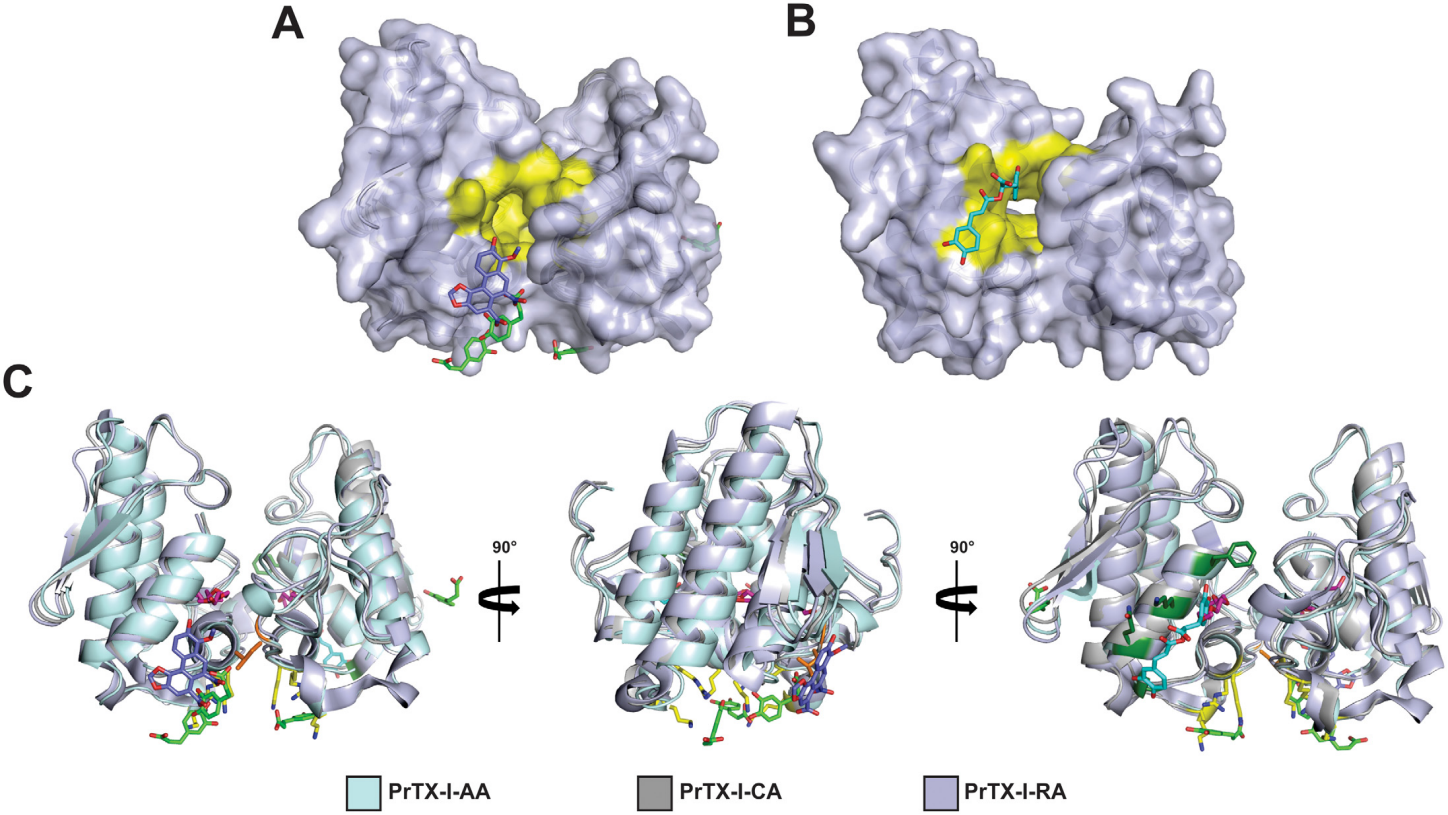
Molecular surface of the PrTX-I complexed to aristolochic acid (PrTX-I/AA) and PrTX-I complexed to caffeic acid (PrTX-I/CA) structures (A) and PrTX-I complexed to rosmarinic acid (PrTX-I/RA) crystal structure (B). PrTX-I/AA and PrTX-I/CA were superimposed using their C^α^ atoms. The region that composes the hydrophobic channel are highlighted in yellow on molecular surfaces. (C)Structural comparison of binding mode of AA, CA, and RA on the PrTX-I structure. PrTX-I/AA, PrTX-I/CA and PrTX-I/RA weresuperimposed using their C^α^ atoms. AA, CA, RA and PEG are highlighted in blue, green, cyan and magenta sticks, respectively. The aminoacidsthat compose MDiS (Leu 121, in orange sticks), MDoS (Lys20, Lys115 and Arg118, in yellow sticks) and hydrophobic channel (Phe3, Lys7, Gln11 and Gly15, in green olivesticks) which interact with AA, CA and RA, respectively, are also highlighted.

AA and CA ligands provided relevant structural information about Lys49-PLA_2_s inhibition towards partial neutralization of the myotoxic activity of these proteins. These ligands experimentally highlighted the previously proposed mechanism of action of Lys49-PLA_2_s, binding with the two interaction sites of these proteins with the targetmembrane (MDoS and MDiS) [18]. However, it is important to note that AA and CA ligands do not inhibitneuromuscular blocking activity. In contrast, RA efficiently inhibits the neuromuscular blocking activity (~90%) and neutralizes approximately ~80% of the myotoxic activity of PrTX-I [47]. The analysis of crystal structure from PrTX-I/RA shows that RA causes the physical blocking of lysophospholipid-binding at the hydrophobic channel, in contrast to PrTX-I/AA and PrTX-I/CA structures, where the hydrophobic channel is completely empty (Fig 7). C^α^superimposition betweenPrTX-I/AA, PrTX-I/CA and PrTX-I/RA structures shows that, in fact, AA, CA and RA bind in different regions of the protein (Fig 7). Furthermore, this superimposition also shows that RA causes the blocking of hydrophobic channel because a catechol group of RA occupies the same regionof part of the PEG molecule in hydrophobic channel present in PrTX-I/AA and PrTX-I/CA structures (Fig 7). Therefore, we can hypothesize thatan inhibitorsuch asRA, which is able toprevent the allosteric transition, aids more efficiently than a ligand (such as AA and CA) that blockades the MDoS and MDiS regions. These datasupport the observation that the binding of AA or CA only can occur efficiently after the allosteric transition, when the MDoS and MDiS regions are exposed to the solvent, with the protein in its active state and able to cause membrane lesion.

The results presentedhere can promote the design of more accurate structure-based inhibitors that, to cause a full inhibition of Lys49-PLA_2_s activity could obstruct both the hydrophobic channel and the MDoS and MDiS regions. The putative drug formed by different inhibitors could obstruct all of these regions simultaneously. Finally, it is important to note that other PLA_2_s-like proteins (e.g., Arg49 and Ser49-PLA_2_s) also have a hydrophobic channel and analogous MDoS and MDiS regions [17]. Consequently, the studies performed here may also be useful to study inhibition methods for these proteins and, consequently, provide an integrated inhibition mechanism for the entire PLA_2_-like protein class.

## Atomic Coordinates

The coordinates were deposited in the Protein Data Bank (PDB) under the identification codes 4YZ7 (PrTX-I/AA) and 4YU7(PrTX-I/CA).
